# Poly[diaqua-μ_2_-isonicotinato-μ_2_-oxalato-terbium(III)]

**DOI:** 10.1107/S1600536808040518

**Published:** 2008-12-20

**Authors:** Wen-Dong Song, Shi-Jie Li, Pei-Wen Qin, Shi-Wei Hu

**Affiliations:** a College of Science, Guang Dong Ocean University, Zhanjiang 524088, People’s Republic of China

## Abstract

In the crystal structure of the title complex, [Tb(C_6_H_4_NO_2_)(C_2_O_4_)(H_2_O)_2_]_*n*_, the Tb^III^ cation is coordinated by four O atoms from two oxalate ligands, two O atoms from two isonicotinate ligands and two O atoms from water mol­ecules within a distorted square–anti­prismatic coordination. The Tb^III^ cation, the isonicotinate anion and the two crystallographically independent water mol­ecules occupy general positions, whereas one of the two crystallographically independent oxalate anions is located on a center of inversion, and the second oxalate anion is located on a twofold rotation axis. The Tb^III^ cations are linked by the oxalate and isonicotinate anions into layers, which are connected *via* inter­molecular hydrogen-bonding and π–π stacking [with centroid-to-centroid distances of 3.509 (2) and 3.343 (3) Å] inter­actions into a three-dimensional network.

## Related literature

For general background on coordination polymers and open-framework materials, see: Yaghi *et al.* (1998 ▶, 2003 ▶); Serre *et al.* (2004 ▶); James (2003 ▶). For related structures, see: Xia *et al.* (2004 ▶); Feng *et al.* (2003 ▶). An independent determination of this structure is reported in the following paper, see: Fang *et al.* (2009 ▶).
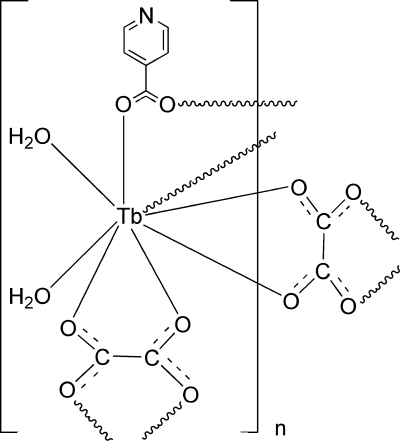


## Experimental

### 

#### Crystal data

[Tb(C_6_H_4_NO_2_)(C_2_O_4_)(H_2_O)_2_]
*M* *_r_* = 405.07
Monoclinic, 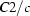

*a* = 17.7957 (6) Å
*b* = 9.9229 (4) Å
*c* = 12.9673 (5) Å
β = 112.407 (2)°
*V* = 2116.95 (14) Å^3^
*Z* = 8
Mo *K*α radiation
μ = 6.72 mm^−1^
*T* = 273 (2) K
0.36 × 0.30 × 0.24 mm

#### Data collection

Bruker APEXII area-detector diffractometer
Absorption correction: multi-scan (*SADABS*; Sheldrick, 1996 ▶) *T*_min_ = 0.113, *T*_max_ = 0.207
7256 measured reflections
1906 independent reflections
1618 reflections with *I* > 2σ(*I*)
*R*_int_ = 0.034

#### Refinement

*R*[*F* ^2^ > 2σ(*F* ^2^)] = 0.023
*wR*(*F* ^2^) = 0.053
*S* = 0.91
1906 reflections
145 parameters
6 restraints
H-atom parameters constrained
Δρ_max_ = 0.52 e Å^−3^
Δρ_min_ = −0.88 e Å^−3^

### 

Data collection: *APEX2* (Bruker, 2004 ▶); cell refinement: *SAINT* (Bruker, 2004 ▶); data reduction: *SAINT*; program(s) used to solve structure: *SHELXS97* (Sheldrick, 2008 ▶); program(s) used to refine structure: *SHELXL97* (Sheldrick, 2008 ▶); molecular graphics: *SHELXTL* (Sheldrick, 2008 ▶); software used to prepare material for publication: *SHELXTL*.

## Supplementary Material

Crystal structure: contains datablocks I, global. DOI: 10.1107/S1600536808040518/nc2122sup1.cif

Structure factors: contains datablocks I. DOI: 10.1107/S1600536808040518/nc2122Isup2.hkl

Additional supplementary materials:  crystallographic information; 3D view; checkCIF report

## Figures and Tables

**Table 1:** Hydrogen-bond geometry (Å, °)

*D*—H⋯*A*	*D*—H	H⋯*A*	*D*⋯*A*	*D*—H⋯*A*
O1*W*—H1*W*⋯N1^i^	0.84	1.83	2.665	177
O1*W*—H2*W*⋯O2^ii^	0.84	2.19	2.992 (3)	159
O2*W*—H3*W*⋯O3^iii^	0.84	2.00	2.835 (3)	177
O2*W*—H4*W*⋯O1*W*^iv^	0.84	2.21	2.998 (3)	156

Symmetry codes: (i) 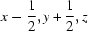; (ii) 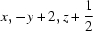; (iii) 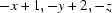; (iv) 
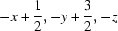
.

**Fig. 1.**
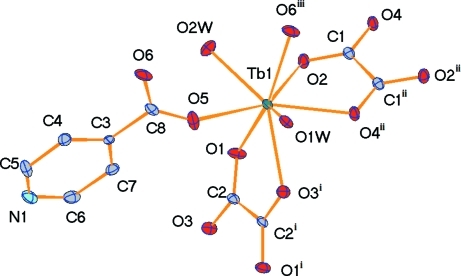
The structure of the title compound, showing the atom-numbering scheme and displacement ellipsoids drawn at the 30% probability level. [Symmetry codes: (i) 1 - x, y, 0.5 - z; (ii) 0.5 - x, 2.5 - y, -z; (iii) 0.5 - x, 1.5 - y, -z]

**Fig. 2.**
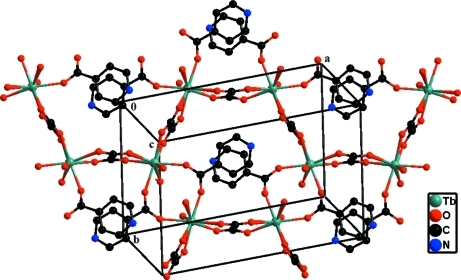
Crystal structure of the title compound with view onto the layers. The H atoms are not shown for clarity.

## supplementary crystallographic information

### Comment

The use of multifunctional organic linker molecules in the preparation of coordination polymers and open-framework materials has led to the development of a rich field of chemistry (Yaghi *et al.*, 1998, 2003; Serre *et al.*, 2004; James, 2003) owing to the potential applications of these materials in catalysis, separation, gas storage and molecular recognition. In our own investigatins we used isonictinate and oxalate ligands for the preparation of new coordination polymers, because it has been found that both anions can act as multidentate ligands [Xia *et al.* (2004); Feng *et al.* (2003)] with versatile binding and coordination modes. During these investigations, single crystals of the title compound were obtained.

The Tb^III^ centre in the title compound exhibits a distorted square-antiprismatic coordination geometry, defined by eight O atoms from two oxalate ligands, two O atom from two isonictinate ligands and two water molecules (Fig. 1). The oxalate and isonictinate ligands link the Tb^III^ cations with Tb—Tb distances of 6.179 (2) Å, 6.183 (3) Å and 5.045 (2) Å, respectively, thus forming Tb-oxalate-isonictinate layers with the attached water that is pointing up and down (Fig. 2). The layers are connected into a three-dimensional network *via* inter/intramolecular O—H···O and O—H···N hydrogen bonding interactions (Table 1) involving the coordinated water molecules, the N atoms of isonictinate and the oxalate O atoms. They are also stabilized by π-π stacking interactions with centroid to centroid distances of 3.509 (2) Å and 3.343 (3) Å, respectively, among parellel pyridinium rings of neighboring complexes.

### Experimental

A mixture of Tb_2_O_3_ (0.5 mmol, 0.175 g), sodium oxalate (1 mmol, 0.134 g), isonicotinic acid (1 mmol, 0.123 g) and H_2_O (10 ml) was placed in a 23 ml Teflon reactor, which was heated to 433 K for three days and then cooled to room temperature at a rate of 10 K h^-1^. The crystals obtained were washed with water and dryed in air.

### Refinement

C-H H atoms were placed in calculated positions and were treated as riding on the parent C atoms with C—H = 0.93 Å, and with *U*_iso_(H) = 1.2 *U*_eq_(C). The O-H H atoms were located in difference Fourier maps and were refined with distance restraints of O–H = 0.84 Å and H···H = 1.39 Å, each within a standard deviation of 0.01 Å, and with *U*_iso_(H) = 1.5 *U*_eq_(O).

### Crystal data

**Table:**

[Tb(C_6_H_4_NO_2_)(C_2_O_4_)(H_2_O)_2_]	*F*(000) = 1536
*M**_r_* = 405.07	*D*_x_ = 2.542 Mg m^−^^3^
Monoclinic, *C*2/*c*	Mo *K*α radiation, λ = 0.71073 Å
Hall symbol: -C 2yc	Cell parameters from 8000 reflections
*a* = 17.7957 (6) Å	θ = 1.7–26.0°
*b* = 9.9229 (4) Å	µ = 6.72 mm^−^^1^
*c* = 12.9673 (5) Å	*T* = 273 K
β = 112.407 (2)°	Block, colorless
*V* = 2116.95 (14) Å^3^	0.36 × 0.30 × 0.24 mm
*Z* = 8	

### Data collection

**Table:**

Bruker APEXII area-detector diffractometer	1906 independent reflections
Radiation source: fine-focus sealed tube	1618 reflections with *I* > 2σ(*I*)
graphite	*R*_int_ = 0.034
φ and ω scans	θ_max_ = 25.2°, θ_min_ = 2.4°
Absorption correction: multi-scan (*SADABS*; Sheldrick, 1996)	*h* = −21→20
*T*_min_ = 0.113, *T*_max_ = 0.207	*k* = −11→11
7256 measured reflections	*l* = −15→15

### Refinement

**Table:**

Refinement on *F*^2^	Primary atom site location: structure-invariant direct methods
Least-squares matrix: full	Secondary atom site location: difference Fourier map
*R*[*F*^2^ > 2σ(*F*^2^)] = 0.023	Hydrogen site location: inferred from neighbouring sites
*wR*(*F*^2^) = 0.053	H-atom parameters constrained
*S* = 0.91	*w* = 1/[σ^2^(*F*_o_^2^) + (0.0299*P*)^2^ + 5.163*P*] where *P* = (*F*_o_^2^ + 2*F*_c_^2^)/3
1906 reflections	(Δ/σ)_max_ = 0.001
145 parameters	Δρ_max_ = 0.52 e Å^−^^3^
6 restraints	Δρ_min_ = −0.88 e Å^−^^3^

### Special details

**Table:**

Geometry. All e.s.d.'s (except the e.s.d. in the dihedral angle between two l.s. planes) are estimated using the full covariance matrix. The cell e.s.d.'s are taken into account individually in the estimation of e.s.d.'s in distances, angles and torsion angles; correlations between e.s.d.'s in cell parameters are only used when they are defined by crystal symmetry. An approximate (isotropic) treatment of cell e.s.d.'s is used for estimating e.s.d.'s involving l.s. planes.
Refinement. Refinement of *F*^2^ against ALL reflections. The weighted *R*-factor *wR* and goodness of fit *S* are based on *F*^2^, conventional *R*-factors *R* are based on *F*, with *F* set to zero for negative *F*^2^. The threshold expression of *F*^2^ > σ(*F*^2^) is used only for calculating *R*-factors(gt) *etc*. and is not relevant to the choice of reflections for refinement. *R*-factors based on *F*^2^ are statistically about twice as large as those based on *F*, and *R*- factors based on ALL data will be even larger.

### Fractional atomic coordinates and isotropic or equivalent isotropic displacement parameters (Å^2^)

**Table:**

	*x*	*y*	*z*	*U*_iso_*/*U*_eq_	
Tb1	0.323989 (12)	0.96756 (2)	0.074846 (17)	0.01399 (9)	
O1	0.46185 (19)	1.0185 (4)	0.1051 (3)	0.0274 (9)	
O2	0.3030 (2)	1.1497 (3)	−0.0596 (3)	0.0229 (8)	
O5	0.3693 (2)	0.7501 (4)	0.1067 (3)	0.0331 (9)	
C1	0.2585 (3)	1.2447 (5)	−0.0542 (4)	0.0172 (11)	
C2	0.5146 (3)	1.0111 (5)	0.2013 (4)	0.0172 (11)	
C7	0.5237 (3)	0.6498 (6)	0.1361 (4)	0.0231 (12)	
H7	0.5190	0.7432	0.1348	0.028*	
C8	0.3727 (3)	0.6297 (6)	0.0808 (4)	0.0196 (11)	
C3	0.4558 (3)	0.5688 (5)	0.1108 (3)	0.0163 (11)	
C6	0.5989 (3)	0.5889 (7)	0.1636 (4)	0.0281 (13)	
H6	0.6444	0.6439	0.1807	0.034*	
C4	0.4671 (3)	0.4306 (5)	0.1135 (4)	0.0220 (12)	
H4	0.4228	0.3731	0.0961	0.026*	
C5	0.54429 (8)	0.37835 (15)	0.14206 (11)	0.0281 (13)	
H5	0.5508	0.2853	0.1440	0.034*	
N1	0.60972 (8)	0.45622 (15)	0.16691 (11)	0.0279 (11)	
O3	0.59033 (8)	1.00505 (15)	0.22872 (11)	0.0229 (8)	
O4	0.22609 (8)	1.33232 (15)	−0.12763 (11)	0.0232 (8)	
O6	0.31250 (8)	0.55572 (15)	0.03267 (11)	0.0301 (9)	
O1W	0.25866 (8)	0.87408 (15)	0.19413 (11)	0.0208 (8)	
H1W	0.2122	0.9030	0.1852	0.031*	
H2W	0.2833	0.8622	0.2630	0.031*	
O2W	0.33025 (8)	0.88114 (15)	−0.09693 (11)	0.0286 (9)	
H3W	0.3550	0.9157	−0.1341	0.043*	
H4W	0.2966	0.8264	−0.1394	0.043*	

### Atomic displacement parameters (Å^2^)

**Table:**

	*U*^11^	*U*^22^	*U*^33^	*U*^12^	*U*^13^	*U*^23^
Tb1	0.01048 (13)	0.01587 (15)	0.01480 (12)	0.00208 (11)	0.00389 (9)	0.00099 (11)
O1	0.0140 (17)	0.047 (3)	0.0180 (17)	−0.0045 (17)	0.0027 (14)	0.0043 (17)
O2	0.0294 (19)	0.023 (2)	0.0202 (17)	0.0082 (17)	0.0143 (15)	0.0022 (16)
O5	0.039 (2)	0.028 (2)	0.039 (2)	0.017 (2)	0.0227 (19)	0.0083 (19)
C1	0.013 (2)	0.019 (3)	0.021 (3)	0.001 (2)	0.007 (2)	0.001 (2)
C2	0.009 (2)	0.017 (3)	0.021 (2)	−0.002 (2)	0.002 (2)	0.000 (2)
C7	0.019 (3)	0.026 (3)	0.022 (3)	−0.004 (2)	0.005 (2)	−0.009 (2)
C8	0.020 (3)	0.030 (3)	0.012 (2)	0.008 (2)	0.008 (2)	0.010 (2)
C3	0.017 (2)	0.024 (3)	0.007 (2)	0.005 (2)	0.0035 (18)	−0.002 (2)
C6	0.016 (3)	0.047 (4)	0.022 (3)	−0.004 (3)	0.008 (2)	−0.006 (3)
C4	0.020 (3)	0.022 (3)	0.023 (3)	−0.002 (2)	0.007 (2)	−0.004 (2)
C5	0.028 (3)	0.026 (3)	0.034 (3)	0.013 (3)	0.016 (2)	0.007 (3)
N1	0.015 (2)	0.046 (4)	0.024 (2)	0.007 (2)	0.0081 (18)	0.003 (2)
O3	0.0175 (18)	0.033 (2)	0.0197 (17)	−0.0024 (16)	0.0089 (14)	0.0018 (15)
O4	0.032 (2)	0.020 (2)	0.0195 (17)	0.0121 (17)	0.0116 (15)	0.0052 (16)
O6	0.0095 (17)	0.050 (3)	0.0259 (18)	0.0005 (18)	0.0013 (14)	0.0006 (18)
O1W	0.0149 (16)	0.028 (2)	0.0201 (16)	0.0064 (16)	0.0076 (14)	0.0074 (16)
O2W	0.040 (2)	0.028 (2)	0.0232 (18)	−0.0068 (19)	0.0179 (17)	−0.0074 (17)

### Geometric parameters (Å, °)

**Table:**

Tb1—O5	2.285 (4)	C7—H7	0.9300
Tb1—O6^i^	2.3055 (14)	C8—O6	1.251 (5)
Tb1—O4^ii^	2.3814 (14)	C8—C3	1.505 (6)
Tb1—O1	2.386 (3)	C3—C4	1.385 (7)
Tb1—O2W	2.4286 (13)	C6—N1	1.329 (7)
Tb1—O2	2.439 (3)	C6—H6	0.9300
Tb1—O1W	2.4444 (13)	C4—C5	1.380 (5)
Tb1—O3^iii^	2.4476 (13)	C4—H4	0.9300
O1—C2	1.245 (6)	C5—N1	1.3306
O2—C1	1.251 (5)	C5—H5	0.9300
O5—C8	1.249 (6)	O3—Tb1^iii^	2.4476 (13)
C1—O4	1.255 (5)	O4—Tb1^ii^	2.3814 (14)
C1—C1^ii^	1.549 (9)	O6—Tb1^i^	2.3055 (14)
C2—O3	1.257 (5)	O1W—H1W	0.8400
C2—C2^iii^	1.537 (9)	O1W—H2W	0.8400
C7—C3	1.382 (7)	O2W—H3W	0.8400
C7—C6	1.387 (7)	O2W—H4W	0.8400
			
O5—Tb1—O6^i^	103.43 (11)	O4—C1—C1^ii^	116.8 (5)
O5—Tb1—O4^ii^	151.65 (9)	O1—C2—O3	127.1 (4)
O6^i^—Tb1—O4^ii^	80.4	O1—C2—C2^iii^	117.5 (5)
O5—Tb1—O1	84.30 (13)	O3—C2—C2^iii^	115.4 (5)
O6^i^—Tb1—O1	154.26 (9)	C3—C7—C6	118.6 (5)
O4^ii^—Tb1—O1	104.49 (10)	C3—C7—H7	120.7
O5—Tb1—O2W	72.32 (10)	C6—C7—H7	120.7
O6^i^—Tb1—O2W	79.4	O5—C8—O6	125.1 (4)
O4^ii^—Tb1—O2W	135.3	O5—C8—C3	117.3 (5)
O1—Tb1—O2W	79.74 (9)	O6—C8—C3	117.6 (4)
O5—Tb1—O2	140.94 (11)	C7—C3—C4	117.7 (4)
O6^i^—Tb1—O2	78.63 (9)	C7—C3—C8	120.8 (5)
O4^ii^—Tb1—O2	67.37 (8)	C4—C3—C8	121.5 (4)
O1—Tb1—O2	80.13 (11)	N1—C6—C7	123.6 (5)
O2W—Tb1—O2	69.77 (8)	N1—C6—H6	118.2
O5—Tb1—O1W	75.35 (9)	C7—C6—H6	118.2
O6^i^—Tb1—O1W	72.49 (5)	C5—C4—C3	120.0 (4)
O4^ii^—Tb1—O1W	79.29 (5)	C5—C4—H4	120.0
O1—Tb1—O1W	133.11 (8)	C3—C4—H4	120.0
O2W—Tb1—O1W	130.20 (6)	N1—C5—C4	122.4 (2)
O2—Tb1—O1W	138.86 (8)	N1—C5—H5	118.8
O5—Tb1—O3^iii^	85.32 (10)	C4—C5—H5	118.8
O6^i^—Tb1—O3^iii^	137.68 (6)	C6—N1—C5	117.7 (2)
O4^ii^—Tb1—O3^iii^	74.2	C2—O3—Tb1^iii^	118.5 (2)
O1—Tb1—O3^iii^	66.61 (8)	C1—O4—Tb1^ii^	118.2 (2)
O2W—Tb1—O3^iii^	141.3	C8—O6—Tb1^i^	149.8 (3)
O2—Tb1—O3^iii^	119.75 (9)	Tb1—O1W—H1W	117.6
O1W—Tb1—O3^iii^	69.9	Tb1—O1W—H2W	123.0
C2—O1—Tb1	119.2 (3)	H1W—O1W—H2W	106.4
C1—O2—Tb1	116.5 (3)	Tb1—O2W—H3W	126.8
C8—O5—Tb1	154.8 (3)	Tb1—O2W—H4W	124.0
O2—C1—O4	126.6 (4)	H3W—O2W—H4W	106.6
O2—C1—C1^ii^	116.6 (5)		

Symmetry codes: (i) −*x*+1/2, −*y*+3/2, −*z*; (ii) −*x*+1/2, −*y*+5/2, −*z*; (iii) −*x*+1, *y*, −*z*+1/2.

### Hydrogen-bond geometry (Å, °)

**Table:**

*D*—H···*A*	*D*—H	H···*A*	*D*···*A*	*D*—H···*A*
O1W—H1W···N1^iv^	0.84	1.83	2.665	177
O1W—H2W···O2^v^	0.84	2.19	2.992 (3)	159
O2W—H3W···O3^vi^	0.84	2.00	2.835 (3)	177
O2W—H4W···O1W^i^	0.84	2.21	2.998 (3)	156

Symmetry codes: (iv) *x*−1/2, *y*+1/2, *z*; (v) *x*, −*y*+2, *z*+1/2; (vi) −*x*+1, −*y*+2, −*z*; (i) −*x*+1/2, −*y*+3/2, −*z*.
